# Molecular basis of ‘*hypoxic*’ breast cancer cell radio-sensitization: phytochemicals converge on radiation induced Rel signaling

**DOI:** 10.1186/1748-717X-8-46

**Published:** 2013-03-04

**Authors:** Sheeja Aravindan, Mohan Natarajan, Terence S Herman, Vibhudutta Awasthi, Natarajan Aravindan

**Affiliations:** 1 Department of Radiation Oncology, University of Oklahoma Health Sciences Center, Oklahoma City, OK, USA; 2 Department of Pathology, University of Texas Health Sciences Center, San Antonio, TX, USA; 3 Department of Pharmaceutical Sciences, University of Oklahoma Health Sciences Center, 800 NE 10th Street, Oklahoma City, OK, USA

**Keywords:** Breast cancer, Hypoxia, Radio-sensitization, Phytochemicals, NFκB, Curcumin, EF24, Neem leaf extract, Genistein, Resveratrol, Raspberry extract

## Abstract

**Background:**

Heterogeneously distributed hypoxic areas are a characteristic property of locally advanced breast cancers (BCa) and generally associated with therapeutic resistance, metastases, and poor patient survival. About 50% of locally advanced BCa, where radiotherapy is less effective are suggested to be due to hypoxic regions. In this study, we investigated the potential of bioactive phytochemicals in radio-sensitizing hypoxic BCa cells.

**Methods:**

Hypoxic (O2-2.5%; N2-92.5%; CO2-5%) MCF-7 cells were exposed to 4 Gy radiation (IR) alone or after pretreatment with Curcumin (CUR), curcumin analog EF24, neem leaf extract (NLE), Genistein (GEN), Resveratrol (RES) or raspberry extract (RSE). The cells were examined for inhibition of NFκB activity, transcriptional modulation of 88 NFκB signaling pathway genes, activation and cellular localization of radio-responsive NFκB related mediators, eNos, Erk1/2, SOD2, Akt1/2/3, p50, p65, pIκBα, TNFα, Birc-1, -2, -5 and associated induction of cell death.

**Results:**

EMSA revealed that cells exposed to phytochemicals showed complete suppression of IR-induced NFκB. Relatively, cells exposed EF24 revealed a robust inhibition of IR-induced NFκB. QPCR profiling showed induced expression of 53 NFκB signaling pathway genes after IR. Conversely, 53, 50, 53, 53, 53 and 53 of IR-induced genes were inhibited with EF24, NLE, CUR, GEN, RES and RSE respectively. In addition, 25, 29, 24, 16, 11 and 21 of 35 IR-suppressed genes were further inhibited with EF24, NLE, CUR, GEN, RES and RSE respectively. Immunoblotting revealed a significant attenuating effect of IR-modulated radio-responsive eNos, Erk1/2, SOD2, Akt1/2/3, p50, p65, pIκBα, TNFα, Birc-1, -2 and −5 with EF24, NLE, CUR, GEN, RES or RSE. Annexin V-FITC staining showed a consistent and significant induction of IR-induced cell death with these phytochemicals. Notably, EF24 robustly conferred IR-induced cell death.

**Conclusions:**

Together, these data identifies the potential hypoxic cell radio-sensitizers and further implies that the induced radio-sensitization may be exerted by selectively targeting IR-induced NFκB signaling.

## Background

Radiotherapy (RT) remains one of the prime treatment modality for breast cancer. It was established mainly in the 1990s that a low pre-treatment intra-tumoral partial oxygen pressure (pO2), as determined by needle electrode measurement, is associated with a poor outcome of treatment, in particular RT but also surgical treatment of several tumor types [1,2]. This association has been explained by the reduced ability of ionizing radiation (IR) to produce DNA damage in the absence of oxygen as well as, more recently, by an increased potential of hypoxic tumor cells for proliferation, invasion, metastasis and angiogenesis [3]. Consistently, studies have determined that poor tumor oxygenation is the strongest prognostic indicator of radiotherapy treatment outcome [4-6] and have indicated that breast tumor pO_2_ distribution prior to radiotherapy is sufficient to predict local response [7].

Tissue hypoxia, an imbalance between oxygen delivery and oxygen consumption resulting in the reduction of oxygen tension below the normal level for a specific tissue [8], is a common feature of most solid tumors including breast carcinoma. This is partly due to the abnormal vascularization in tumors, which is insufficient in supplying O_2_ to the sometimes rapidly expanding malignant lesions. The use of polarographic O_2_ needle electrodes have revealed low pO_2_ values of 23–28 mmHg (median) for tumors, as opposed to benign lesions (42 mmHg) and normal tissue (54–65 mmHg) [9,10]. Particularly, of all readings taken from breast cancers, 30–40% falls below 10 mmHg, which is very rarely seen in normal tissue [9,10]. Further, studies have revealed that nearly 40% of breast malignancies exhibit tumor regions with oxygen concentrations below that required for half-maximal radio-sensitivity (pO_2_ < 2.5 mmHg) [9]. To that end, for decades, investigators have attempted to overcome the treatment resistance of hypoxic tumors in clinical trials, for example by adding so-called 'hypoxic radio-sensitizer' drugs to the regimens or introducing hyperbaric oxygen. Although many of the individual trials were negative, a modern meta-analysis confirms the efficacy of hypoxia-directed treatments [11]. Consequently, recent research focusing on hypoxic responses and delineation of important regulators of hypoxia has clearly indicated that ‘the hypoxia response’ in tumors can be used to define novel treatment strategies [8]. The future arsenal of novel cancer therapies will most certainly include specific targeting of hypoxic process.

The presence and extent of the hypoxic tumor microenvironments have recently been shown to influence tumor progression by regulating both tumor cell survival and the expression of key angiogenic molecules. To that end, numerous studies have implicated the transcription factor, NFκB as a mediator of the hypoxic processes in tumor cells [12,13]. On the other hand, we and others have demonstrated a pronounced activation of NFκB in response to IR in tumor cells including breast cancer cells [14-17]. NFκB is a member of the c-*rel* proto-oncogene family found within the promoter and enhancer region of a wide variety of cellular genes involved in proliferation, differentiation and cell cycle control [18,19]. Unlike other inducible transcription factors, a multitude of conditions and agents, including IR, can activate NFκB in cells. On activation, NFκB can stimulate various targeted late-response genes [18,19], including those responsible for cell cycle control, oncogenic activation, [20], cell growth, differentiation and metastasis [21,22]. More importantly, studies have established the influence of NFκB in induced adaptive resistance, inflammatory and proliferation response [23-26]. Together with the fact that NFκB is able to regulate >150 genes, hypoxia induced NFκB signal transduction and activity may play a key role in transcriptional activation of these downstream targets that potentially regulate radio-sensitization.

A plethora of recent research has been focused on exploiting the pharmacologically safe, bioactive phytochemicals as potent radio-sensitizers in a number of tumor types including breast cancer. To that note, we have shown that nutraceuticals viz., curcumin (CUR), black raspberry extract (RSE), neem leaf extract (NLE) etc., regulate a number of potential molecular targets and potentiate radio-sensitization in neuroblastoma, pancreatic cancer and breast tumor systems [15,27-31]. Here in, we investigated the effects of CUR, curcumin analog EF24, NLE, Genistein (GEN), Resveratrol (RES) and RSE on the NFκB DNA-binding activity and NFκB signal transduction in hypoxic breast cancer cells exposed to IR. Furthermore, we elucxidated the effects of these bioactives in the activation and cellular localization of hypoxia-responsive NFκB related effectors including p53, Akt, Nos3, Erk1/2, SOD2, p50, p65, TNFα, IAP1, IAP2 and Survivin. More importantly, we elucidated the efficacy of these compounds in potentiating IR induced hypoxic cell killing in this setting. In hypoxic breast cancer cells, CUR, EF24, NLE, GEN, RES and RSE resulted in the (i) complete suppression of IR-induced NFκB-DNA binding activity (ii) attenuation of IR-induced NFκB signal transduction and target transcriptome, (iii) mitigation of IR-induced Akt,, Nos3, Erk1/2, SOD2, p50, p65, TNFα, Birc 1, 2 and 5 and (iv) potentiates IR-induced cell killing, implying that these bioactive phytochemicals may play a key role in regulating NFκB signaling pathway dependent ‘*hypoxic processes’* and may potentiate RT in this setting.

## Methods

### Cell culture

Estrogen receptor positive human adenocarcinoma (MCF-7) breast cancer cells were maintained as monolayer cultures by weekly serial passage in 100 mm tissue culture plates in Dulbecco’s Modified Eagle medium (Cellgro, Herndon, VA) with 44 mM sodium bi-carbonate, 4 mM L-glutamine, supplemented with 10.2 IU/ml penicillin/10.2 mg/ml streptomycin, and 10% heat-inactivated fetal bovine serum (Atlanta Biological, Lawrence Ville, GA). When in exponential growth, the cells were observed to have a doubling time of approximately 16–18 h. For passage and for all experiments, the cells were detached using trypsin (0.25%)/EDTA (1%), re-suspended in complete medium, counted electronically using Countess Cell counter (Life Technologies Corp.) and incubated in a 95% air/5% CO2 humidified incubator.

### Hypoxia, phytochemicals treatment and Irradiation experiments

The cells plated in 100 mm tissue culture plates containing 6 ml of complete growth medium were allowed to grow up to 70–80% confluence. Then the cells were made quiescent by serum starvation overnight followed by treatment. For growth under hypoxia, the cells were incubated at 37°C in a modular chamber flushed with 2.5% O_2_, 5% CO_2_ and 92.5% N_2_. For phytochemical studies, 2.0μg/ml RSE [27,31], 100nM CUR [15,29,31], 0.1% NLE [30,31], 200nM EF24 [32], 100 μM GEN and 100 μM RES was added to the medium for 1 h before hypoxia. For IR experiments, the cells were irradiated with one single high dose (SDR-4 Gy) using Gamma Cell 40 Exactor (Nordion International Inc, Ontario, Canada) at a dose rate of 0.81 Gy/min. Mock irradiated cells were treated identical except that the cells were not subjected to IR. The experiments were repeated at least three times in each group. In all the groups the cells were harvested 24 h after IR.

### Electrophoretic mobility shift assay

Nuclear protein extraction and electrophoretic mobility shift assay for NFκB, were performed as described in our earlier studies [33]. Autoradiograms were overexposed to portrait the inhibitory effect below constitutive level. Densitometry analysis was performed using a BioRad Multi-Analyst software package with an integrated density program. Group-wise comparisons were made using ANOVA with Tukey’s post-hoc correction. A P value of <0.05 is considered statistically significant. For the competition assay, the nuclear extract was pre-incubated with unlabeled homologous NFκB oligonucleotide followed by addition of [γ-^32^P]-ATP labeled NFκB probe. Super shift analysis was performed as described earlier [33].

### Real-Time QPCR profiling of NFκB signaling pathway molecules

Total RNA extraction and real-time QPCR profiling were performed as described in our earlier studies [33,34]. We used human NFκB signaling pathway profiler (Realtimeprimers.com, Elkins Park, PA) containing 88 genes representing 8 functional groups including (i) Rel/NFκB/IκB family, (ii) NFκB responsive genes, (iii) Ligands & Transmembrane receptors, (iv) Adaptor proteins, (v) Signal transduction kinases, (vi) Transcription factors, (vii) Cell death/survival molecules, and (viii) Other factors. We started with this highly selected QPCR profiler instead of an all-encompassing gene array because the selected genes entail a well-characterized profile governing NFκB signal transduction and transcriptional targets, hence facilitating interpretation of data, simplifying data acquisition and analysis, and avoiding genes not functionally characterized. Furthermore, QPCR profiling allows detection and quantification of gene expression in real-time. Each profiling plate was also equipped with reverse transcription control, positive PCR control, genomic DNA control and five housekeeping genes – *β*-*Actin*, *GAPDH*, *Rpl13a*, *HPRT1* and *β2M*. The ΔΔ^ct^ values were calculated by normalizing the gene expression levels to the expression of the housekeeping genes. The normalized data were then compared between groups, and the relative expression level of each gene was expressed as fold change.

### Immunoblotting

Total protein extraction and immunoblotting were performed as described in our earlier studies [33]. Rabbit polyclonal anti-Birc1, 2, 5, eNOS, SOD2, AKT1/2/3, TNFα, p53, pAKT1/2/3 (all obtained from Santa Cruz Biotechnology Inc., Santa Cruz, CA), p50, p65 (obtained from Abcam, Cambridge, MA), ERK1/2 (Cell Signaling Technology, Danvers, MA), mouse monoclonal anti-pERK1/2 (Cell Signaling), pIκBα (Santa Cruz) and, goat polyclonal anti-peNOS (Santa Cruz) antibodies were used to detect the respective protein expression and phosphorylation levels in response to hypoxia and IR in conjunction with different phytochemicals. Blots were stripped and reprobed with mouse monoclonal anti-α-tubulin antibody (Santa Cruz) to determine equal loading of the samples. 1D gel analysis was performed using a BioRad Multi-Analyst software package with an integrated density program. Group-wise comparisons were made using ANOVA with Tukey’s post-hoc correction. A P value of <0.05 is considered as statistically significant.

### Annexin-V binding assay

The apoptotic events was quantified by the binding of Annexin-V to the externalized protein, phosphatidylserine, on the cell membrane during apoptosis with the ApoScreen Annexin V-Fluorescein (FITC) Kit (Southern Biotech, Birmingham, AL), according to the manufacturer’s instructions. Briefly, the floating and trypsinized-adherent of hypoxic and untreated cells with or without IR and/or phytochemicals were collected and washed with PBS. Cells were resuspended in 1X binding buffer to a concentration of 1x10^7^ cells/ml were then stained with 10 μL of Annexin V-FITC and 5 μl of propidium iodide (50 μg/ml) for 15 min on ice, protected from light. Cells were analyzed using FACS Calibur flow cytometer equipped with a 488 nm argon laser and gated on viable cells by light scatter. Group-wise comparisons were made using ANOVA with Tukey’s post-hoc correction. A P value of <0.05 is considered as statistically significant.

## Results

### Nutraceuticals impede IR-induced p50/p65 expression and DNA-binding activity in hypoxic BCa cells

**Figure 1:**
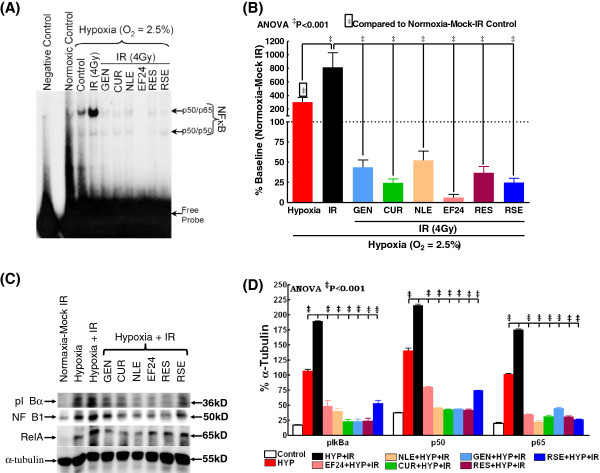
**(A) A representative autoradiogram showing NFκB DNA-binding activity levels in MCF-7 cells exposed to hypoxia and/or radiation with or without EF24, NLE, CUR, GEN, RES or RSE pre-treatment.** The nuclear extracts were analyzed by EMSA using γ-^32^p [ATP] labeled NFκB-specific probe. Compared to the normoxia controls, hypoxic cells showed a significant induction of NFκB-DNA binding activity. IR-exposure further enhanced hypoxia induced NFκB activity. Hypoxic cells treated with EF24, NLE, CUR, GEN, RES or RSE showed a significant inhibition of IR-induced NFκB-DNA binding activity. **(B)** Semi-quantitative densitometric analysis showing the effect of EF24, NLE, CUR, GEN, RES or RSE on IR-induced NFκB-DNA binding activity in hypoxic MCF-7 cells. **(C)** Western blot analysis showing modulation in the IκBα phosphorylation, nuclear translocation of NFκB p50 and p65 in hypoxic MCF-7 cells exposed to IR with or without EF24, NLE, CUR, GEN, RES or RSE treatment. Total cell extracts for pIκBα and nuclear extracts for p50 and p65 were used.α-tubulin expression was determined to validate equal sample loading. **(D)** Histograms of densitometric analysis normalized to α-tubulin expression showing complete inhibition of IR-induced IκBα phosphorylation and nuclear translocation of p50/p65 in hypoxic BCa cells with EF24, NLE, CUR, GEN, RES or RSE treatment. Group-wise comparisons were made using two-way ANOVA with Tukey’s post-hoc correction.

To delineate the effect of IR on NFκB expression and DNA-binding activity in hypoxic breast cancer cells and further to elucidate the efficacy of nutraceuticals in this setting, human BCa (MCF-7) cells cultured under hypoxic conditions were either mock-irradiated or exposed to IR (2 Gy) with or without EF24, NLE, CUR, GEN, RES and RSE pre-treatment and harvested after 24 h post-IR. Compared to cells grown under normoxia, EMSA revealed a significant induction (300.3±71.6%) of NFκB-DNA binding activity in hypoxic BCa cells (Figure 1A). Further, exposure of hypoxic cells to IR resulted in a robust (813.7±216.5) induction of NFκB DNA-binding activity. On the other hand, we observed a consistent and complete inhibition of this IR-induced DNA-binding activity in EF24 (6.2±4.0%), NLE (52.5±11.3%), CUR (24.4±4.9%), GEN (43.8±9.1), RES (36.9±7.7%) and RSE (24.8±5.3%) pre-treated hypoxic MCF-7 cells. More importantly, an assiduous inhibitory potential of these nutraceuticals was evident with NFκB-DNA binding levels lesser than that of normoxic-mock-IR cells (Figure 1B). Consistently, western blot analysis from the nuclear extract revealed a significant induction of NFκB p50 and p65 levels in hypoxic BCa cells (Figure 1C). This hypoxia induced nuclear translocation of p50 and p65 is further enhanced with IR-exposure. More importantly, EF24, NLE, CUR, GEN, RES and RSE significantly reverted the IR-induced p50/p65 nuclear translocation in this setting (Figure 1C & D). On the other hand, we observed an induced IκBα phosphorylation in BCa cells grown under hypoxic conditions, which was further enhanced when the cells were exposed to IR (Figure 1D). However, a consistent inhibition of IκBα phosphorylation in hypoxic cells pre-treated with EF24, NLE, CUR, GEN, RES or RSE and exposed to IR was evident (Figure 1D). These results correlated well with the NFκB data which together validates the regulation of NFκB in response to hypoxia and radiation with or without nutraceuticals.

### Nutraceuticals targets NFκB signaling transcriptome in hypoxic breast cancer cells

**Figure 2:**
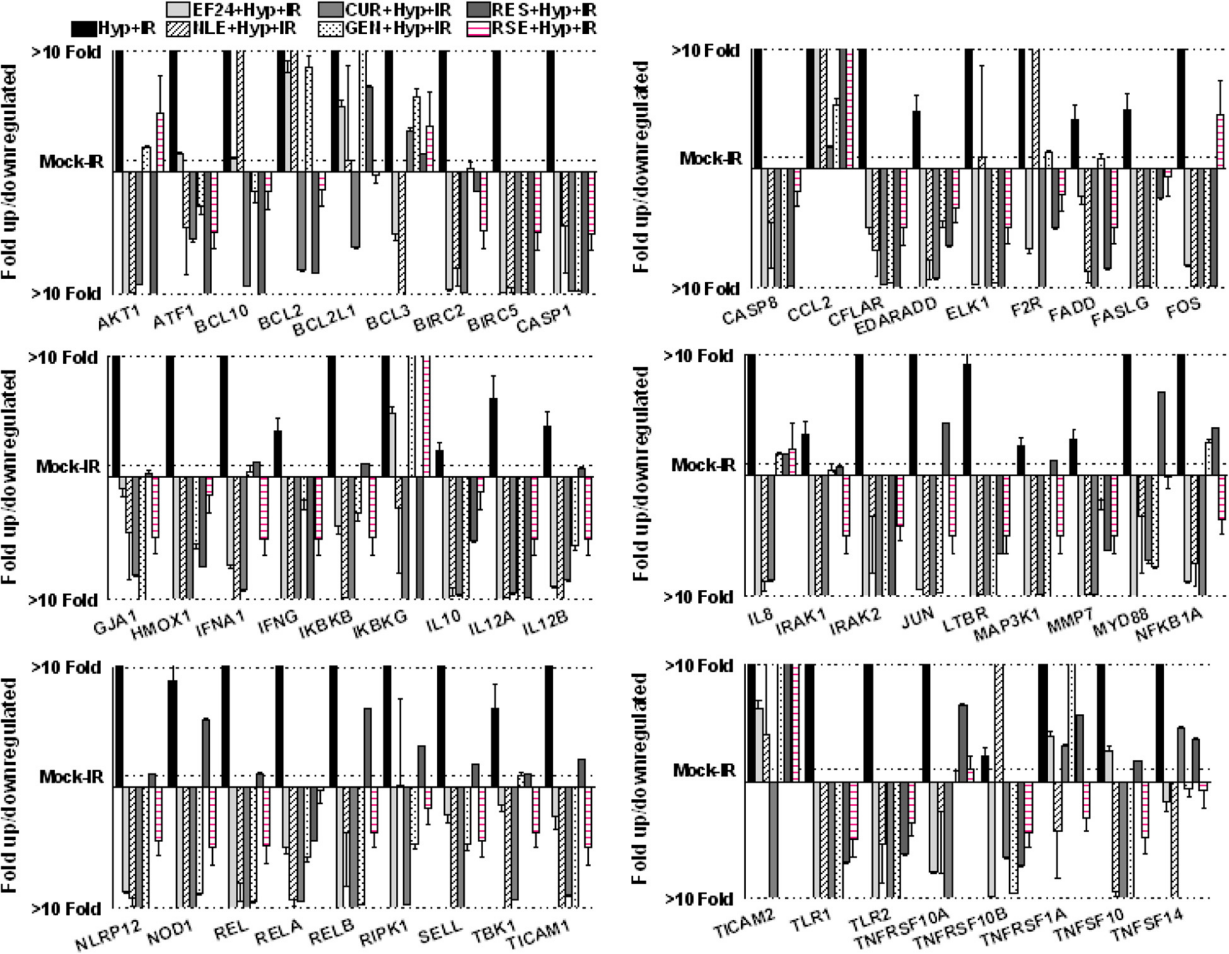
**Histograms showing attenuation and/or complete suppression of 59 IR-induced NFκB signaling pathway genes with CUR, EF24, NLE, GEN, RES or RSE treatment in hypoxic human breast adenocarcinoma cells.** QPCR profiling was performed using human NFκB signaling pathway profiler (Realtimeprimers.com, Elkins Park, PA) containing 88 genes and 8 internal controls. The ΔΔ^ct^ values calculated by normalizing the gene expression levels to the expression of the housekeeping genes were compared between groups and expressed as fold change.

Further to substantiate that bioactive nutraceuticals intervene IR-induced NFκB signaling in hypoxic cells, MCF-7 cells cultured under hypoxic conditions were either mock-irradiated or exposed to IR (2 Gy) with or without EF24, NLE, CUR, GEN, RES and RSE pre-treatment and harvested after 3 h post-IR. Custom QPCR profiling of 88 NFκB upstream/downstream signal transduction pathway molecules revealed that clinically relevant doses of radiation significantly induced 53 genes (Figure 2) and suppressed another 35 genes in hypoxic breast cancer cells. To that end, IR robustly increased the transcription of NFκB family molecules including Rel, RelA, RelB, IKKβ and IKKγ and, serves as the positive controls for the study. Interestingly, presence of nutraceuticals in the system completely and comprehensively reverted this IR-induced NFκB signal transduction in hypoxic cells. Treatment with EF24, CUR, GEN, RES and RSE resulted in complete suppression of all 53 IR-induced NFκB signal transduction genes in these cells under hypoxic conditions. NLE reverted 50 of 53 IR-induced genes in this setting (Figure 2). Evidently, all the nutraceuticals investigated revealed a strong potential for the inhibition of Rel family molecules. Altogether, 50 of 53 IR-induced NFκB signaling pathway genes were targeted in common with EF24, NLE, CUR, GEN, RES and RSE. Conversely, IR-exposure resulted in the inhibition of 35 NFκB signaling pathway genes as opposed to hypoxia alone. Notably, EF24, NLE, CUR, GEN, RES and RSE treatment further conferred 25, 29, 24, 16, 11 and 21 of those IR-suppressed genes. Of these, five genes including *Csf1*, *NF*κ*B2*, *PPM1A*, *RAF1* and *RHOA* were commonly conferred after all nutraceuticals investigated.

### Nutraceuticals reverts IR-altered cellular localization and/or activation of NFκB signaling pathway proteins in hypoxic breast cancer cells

**Figure 3:**
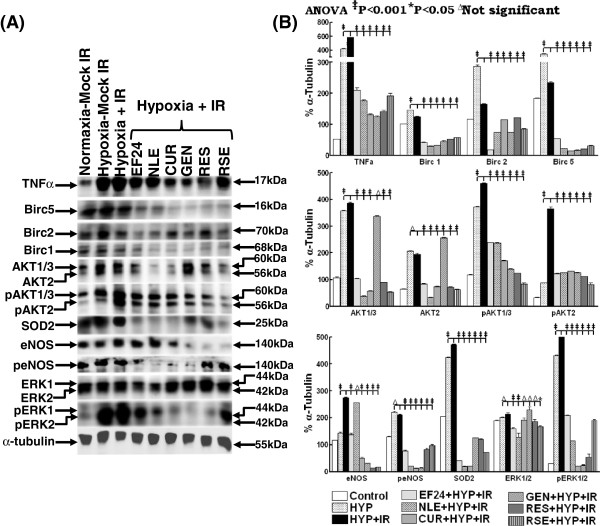
**(A) Western blot analysis showing modulation in TNFα, Birc 1, 2, 5, SOD2, constitutive and phosphorylation of eNOS, AKT1/2/3 and ERK1/2 in hypoxic MCF-7 cells exposed to IR with or without EF24, NLE, CUR, GEN, RES or RSE treatment.** α-tubulin expression was determined to validate equal sample loading. **(B)** Histograms of densitometric analysis normalized to α-tubulin expression showing complete inhibition of hypoxia and IR-induced TNFα, Birc 1, 2, 5, SOD2, and phosphorylation of eNOS, AKT1/2/3 and ERK1/2 in hypoxic BCa cells with EF24, NLE, CUR, GEN, RES or RSE treatment. Group-wise comparisons were made using two-way ANOVA with Tukey’s post-hoc correction.

To confirm further that the bioactive EF24, NLE, CUR, GEN, RES and RSE targeted NFκB signaling pathway transcriptome is in fact translated into the NFκB signal transduction, we examined the alterations in both the cellular levels and activation of certain select NFκB upstream (AKT, ERK, eNOS), downstream (Birc 1, 2, 5) and, bifunctional (TNFα, SOD2) signal transduction/effector proteins (Figure 3A). Compared to normoxic conditions, TNFα and SOD2 immunoblotting revealed a profound increase (P<0.001) in hypoxia treated cells. This hypoxia induced induction of both TNFα and SOD2 was significantly enhanced when the cells were exposed to IR (Figures 3A & B). However, pretreating the cells either with EF24, NLE, CUR, GEN, RES or RSE completely (P<0.001) knock down this hypoxia and/or IR-activated TNFα and SOD2 in human MCF-7 cells (Figure 3B). Consistently, we observed an impetuous increase in NFκB direct downstream targets including Birc 1, 2 and 5 in cells exposed to hypoxia. Radiation on the other hand, significantly reduced the expression of these Bircs in the hypoxic cells. Nevertheless, EF24, NLE, CUR, GEN, RES or RSE treatment in conjunction with IR completely (P<0.001) knocked down these NFκB dependent pro-survival Birc 1, 2 and 5 in hypoxic cells (Figures 3A & B). Analyzing the expression and phosphorylation of NFκB upstream signal transducing AKT, ERK and eNOS conform our transcriptome profiling data. Interestingly, induced expression levels of AKT1/2/3 in response to hypoxia and further with IR exposure were evident in these cells. In parallel, hypoxia in the system instigated a robust (>4 fold) phosphorylation of AKT1/2/3. Notably, this hypoxia induced AKT1/2/3 phosphorylation was further (P<0.001) influenced with IR-exposure. More importantly, EF24, NLE, CUR, RES or RSE completely mute both the IR-induced cellular levels and phosphorylation of AKT1/2/3 in the hypoxic cells (Figures 3A & B). GEN on the other hand showed either no effect or increase in the cellular expression of AKT1/2/3. However, consistent with other bioactives, we observed a significant inhibition of IR-induced AKT1/2/3 phosphorylation in these hypoxic cells. Furthermore, hypoxia resulted in the relative increase in the constitutive eNOS and significant increase in the phosphorylation of eNOS. Interestingly, we did not see any further increase in eNOS phosphorylation with IR exposure. Conversely, EF24, NLE, CUR, GEN, RES or RSE treatment resulted in complete suppression of the hypoxia and/or IR altered eNOS phosphorylation (Figures 3A & B). In addition, we observed a significant and robust increase in the ERK1/2 phosphorylation after hypoxia. Radiation evidently influenced further phosphorylation of ERK1/2 in these hypoxic cells. Consistent with the AKT and eNOS phosphorylation muting observed, we observed a total inhibition of ERK1/2 phosphorylation in EF24, NLE, CUR, GEN, RES or RSE treated cells (Figures 3A & B).

### EF24, NLE, CUR, GEN, RES and RSE confers IR-induced cell death in hypoxic breast cancer cells

**Figure 4:**
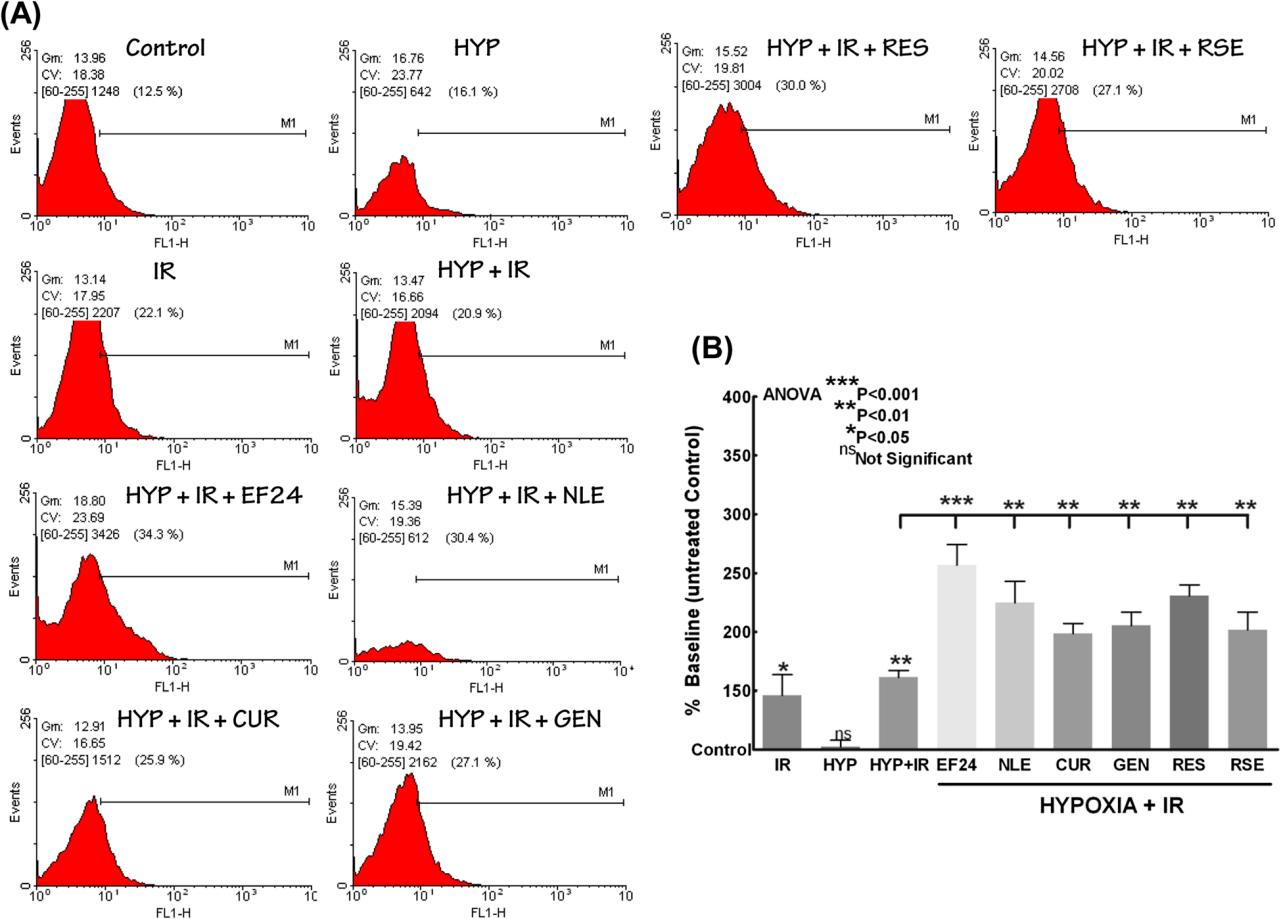
**(A) FACS analysis showing annexin V**-**FITC staining (with ApoScreen Annexin V**-**Fluorescein) in cells under normoxic and hypoxic conditions exposed to IR with or without EF24, NLE, CUR, GEN, RES or RSE treatment. (B)** Histograms showing significant increase in cell killing with IR exposure in hypoxic cells and further radiopotentiating effect of bioactive EF24, NLE, CUR, GEN, RES or RSE in human breast adenocarcinoma (MCF-7) cells exposed to hypoxia. Group-wise comparisons were made using ANOVA with Tukey’s post-hoc correction.

To determine whether the bioactives targeting NFκB signaling cascade is in fact translates to functional response and to elucidate the radio-potentiating effect of these drugs in hypoxic conditions, we examined the alterations in induced cell death in hypoxic cells exposed to IR with or without EF24, NLE, CUR, GEN, RES or RSE treatment (Figure 4A & B). Opposed to mock-IR hypoxic cells, IR exposure significantly induced (P<0.01) cell death in cells under hypoxia. However, coherent with our NFκB data, this IR-induced cell death is profoundly influenced in cells treated with EF24, NLE, CUR, GEN, RES or RSE (Figure 4B). Relatively, novel synthetic monoketone of curcumin, EF24 instigate more radiopotentiating effect as evident with the robust (P<0.001) cell death.

## Discussion

Radiotherapy is very effective in local control of cancerous tumors, but its curative potential is often limited by intrinsic radio-resistance of the tumor cells. Hypoxia is one such limiting factor and indeed a main factor in most tumors including breast cancer that affects the radio-therapeutic ratio. Tumors tend to outgrow their blood supplies and develop hypoxic regions that preferentially protect tumor cells from radiation-induced DNA damage, as oxygen not only fixates DNA damage but also increases the number and complexity of DNA lesions produced by radiation. It has been shown that hypoxia can alter the biological state of tumor cells, including the transcription and translation of various proteins involved in cell survival [35]. Variety of metabolic stress signals including hypoxia activates NFκB [13,36], that rapidly transduce hypoxic signals and facilitates the rapid control of many genes. Mechanisms involved in hypoxia induced NFκB is discussed in detailed elsewhere [37]. Since hypoxia can induce NFκB activation, this pathway may contribute to a pro-malignant phenotype not only by promoting cell proliferation and survival, but also by upregulating gene products that control cell adhesion and angiogenesis. To that note, though a number of studies have identified the possible role of NFκB dependent downstream response in hypoxic processes, a definitive insight on the regulation of NFκB signaling in response to hypoxia in breast cancer, if not in any tumors is still at vague. Moreover, how this hypoxia associated NFκB response translates after any prime therapeutic intervention is not understood. To our knowledge, this is the first attempt to characterize NFκB signaling in hypoxic breast cancer cells in response to a treatment modality, here in this case, radiotherapy and, further to delineate the modulation of altered signaling response to known anti-tumor phytochemicals. Consistent with the published evidence [13,36,38], hypoxia resulted in significant induction of NFκB activity and serves as the positive controls for the study. In addition, the results also add on a new insight on the altered NFκB signaling pathway transcriptome modulation in response to hypoxia in these cells. However, for the first time, this study identified that the radiation instigated pronounced NFκB activation and its signal transduction in surviving hypoxic breast cancer cells. Both from our laboratory and studies from other investigators demonstrated that clinical radiotherapy resulted in predominant activation of NFκB in surviving tumor cells including breast cancer cells [15-17,27-29,31,33,39]. The results presented here portraits that radiation robustly increased nuclear translocation and DNA binding activity of NFκB, transactivation of 53 NFκB family, signaling and effector molecules and further resulted in activation of TNFα, AKT1/2/3, SOD2 and ERK1/2 at least in surviving hypoxic breast cancer cells. A plethora of evidence has demonstrated that both AKT and ERK are upstream of NFκB and are actively involved in NFκB activation. Like-wise, we have demonstrated that TNFα and SOD2 though are the direct downstream targets of NFκB signaling are in fact individually exerts a positive feedback input with NFκB signaling that promotes robust and persistent NFκB activation after radiation [33,34].

**Figure 5:**
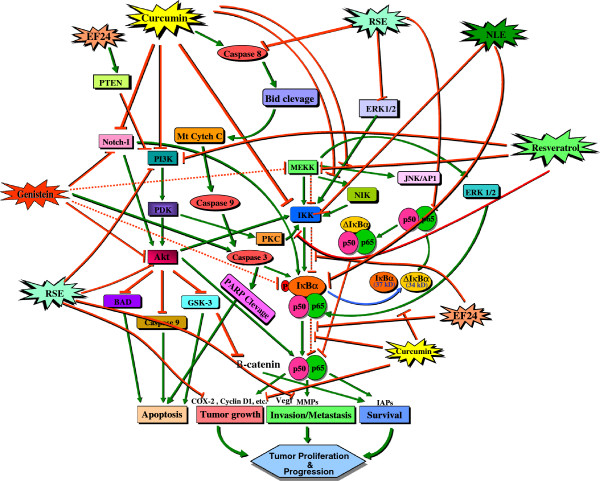
**Schematic illustration of phytochemicals targeting NFkB signaling pathway.** Curcumin, EF24, Resveratrol and Genistein targets more than one signal transduction molecules in NFkB pathway and comprehensively affect NFkB dependent tumor progression.

Further, this study, for the first time unveils the understanding of how the known bioactive phytochemicals NLE, CUR, GEN, RES or RSE and synthetic analogue (EF24, in this case) radio-sensitize and/or radio-potentiate hypoxic breast cancer cells. The results precisely demonstrated that phytochemicals including neem leaf extract, genistein, resveratrol, raspberry extract, curcumin and, its synthetic analogue EF24 have significant impact on attenuating radiation modulated NFκB nuclear import, DNA-binding activity, signaling pathway transcriptome and on the translational activation of its signaling (TNFα, AKT1/2/3, SOD2 and ERK1/2) and effector (Birc 1, 2, 5) proteins in surviving hypoxic breast cancer cells. To that end, the significant reverting effect (53 off 53 except NLE where it is 50/53) IR-induced transcriptional response demonstrate for the fact all these compounds converge on the NFκB signaling pathway to inhibit hypoxia induced radio-adaptation and to radio-potentiate cell killing. A simplified schematic pathway map is presented in Figure 5 in an attempt to give a glimpse of NFκB signaling targeted by these compounds. CUR, a dietary polyphenol derived from turmeric, *Curcuma longa*, is a pharmacologically safe and effective agent that has been demonstrated to have anti-inflammatory, antiproliferative, and antitumor effects by modulating many potential molecular targets (47). Because of its use as a food additive and its potential for cancer chemoprevention, curcumin has undergone extensive toxicological screening and pre-clinical investigation in rats, mice, dogs, and monkeys (48). Curcumin has been demonstrated to inhibit NFκB by suppressing the activation of IKK by interacting directly with the kinase (47). Similarly, RES, a phytoalexin present in grape skins and red wines, exerts striking inhibitory effects on diverse cellular events associated with tumor initiation, promotion, and progression [40]. A number of studies showed RES associated inhibition of NFκB and further have dissected out that it exerts the response through (a) phosphorylation/degradation of IκBα via IKK [41]; (b) inhibiting IKK activity [42] or (c) phosphorylation/nuclear translocation of the p65 [43]. Similarly, GEN, the most abundant isoflavone found in soybeans, is believed to be a potent anticancer agent [44,45] and is currently under 34 (7 studies still open) clinical trials (www.clinicaltrials.gov). Notably, NFκB inhibitory effect [46-55] radio-sensitizing effects [55-62] of Genistein have been well documented. To that end, Sarkar and colleagues suggested that Genistein inhibited MEKK1 kinase activity may be responsible for the decreased phosphorylation of IκBα and thereby result in the inactivation of NFκB [63-66]. Furthermore, synthetic analogue of curcumin, EF24 has shown to possess potent anticancer activity, both *in vitro* as well as *in vivo*[67]. More importantly, EF24 has been shown to directly inhibit IKKβ kinase activity [68]. In addition, we have recently shown that bioactive RSE and NLE selectively target radiation induced NFκB in surviving cancer cells and potentiate radiation induced cell death [27,30,31].

In the context of the breast cancer cure, research around the globe is primarily and appropriately focused on dissecting out the potential benefit of bioactive phytochemicals. Indeed, half of the top anticancer drugs in the USA are derived directly or indirectly from natural products [69,70]. To that end, we and others have shown that CUR, NLE, RSE, RES, GEN possess potent anti-breast cancer properties by modulating many potential molecular targets and thereby exerts breast cancer cell killing, prevent tumor growth, invasion and metastasis [27]. Interestingly, studies have shown that at least in part, the benefits of these phytochemicals are imparted by selectively targeting NFκB in breast cancer cells [71-75]. However, as discussed above, hypoxia is a frequent phenomenon in breast cancer, orchestrated as a result of tumor outgrowing the existing vasculature leading to an adaptive response. Radiation as one of the prime treatment modality for breast cancer, hypoxia significantly affects the radio-therapeutic ratio in breast cancer. Our result in the present study has shown that the cellular response of hypoxia is modulated by the ubiquitous transcription factor NFκB. Besides the fact that hypoxia affects general radio-sensitization processes such as apoptosis and proliferation, our data linked hypoxia associated NFκB activation to the radio-protective phenotype in breast cancer cells. Exquisitely, the data presented here demonstrated the potential of CUR, EF24, NLE, RSE, RES and GEN in targeting not only radiation-induced NFκB activation but also NFκB signal transduction and downstream NFκB dependent translational/functional response. Though a lot need to be explored on response initiation signaling and molecular mechanism(s) upstream of NFκB by these phytochemicals, this study, *per se* ostensibly identified that curcumin, its analogue EF24, genistein, resveratrol, raspberry extract and neem leaf extract converge on selective inhibition of NFκB signaling and thereby potentiates radiotherapy in hypoxic breast cancer cells. Further studies delineating the drug and radiation dose, functional downstream molecular (effector) orchestration, pre-clinical efficacy with appropriate animal models, multi-compound combinatorial synergism, if any, are warranted and are currently pursued in our laboratory.

## Conclusions

In this study, we investigated the effects of CUR, curcumin analog EF24, NLE, GEN, RES and RSE on the NFκB DNA-binding activity and NFκB signal transduction in hypoxic breast cancer cells exposed to IR. Furthermore, we elucidated the effects of these bioactives in the activation and cellular localization of hypoxia-responsive NFκB related effectors including p53, Akt, Nos3, Erk1/2, SOD2, p50, p65, TNFα, IAP1, IAP2 and Survivin. More importantly, we elucidated the efficacy of these compounds in potentiating IR induced hypoxic cell killing in this setting. In hypoxic breast cancer cells, CUR, EF24, NLE, GEN, RES and RSE resulted in a (i) a complete suppression of IR-induced NFκB-DNA binding activity (ii) attenuation of IR-induced NFκB signal transduction and target transcriptome, (iii) mitigation of IR-induced Akt,, Nos3, Erk1/2, SOD2, p50, p65, TNFα, Birc 1, 2 and 5 and (iv) potentiates IR-induced cell killing, implying that these bioactive phytochemicals may play a key role in regulating NFκB signaling pathway dependent ‘*hypoxic processes*’ and may potentiate R T in this setting.

## Competing interests

The authors declare that they have no competing interests.

## Authors’ contributions

SA carried out the molecular assays and helped to draft the manuscript. MN participated in the design of the study and performed the statistical analysis. TSH participated in the design of the study and helped to draft the manuscript. VA carried out the EF24 synthesis, NLE and RSE characterization. NA conceived the study, and participated in its design and coordination and drafted the manuscript. All authors read and approved the final manuscript.
